# Preparation of silica nanospheres and porous polymer membranes with controlled morphologies via nanophase separation

**DOI:** 10.1186/1556-276X-7-440

**Published:** 2012-08-08

**Authors:** Jung-Pil Lee, Sinho Choi, Soojin Park

**Affiliations:** 1 Interdisciplinary School of Green Energy, Ulsan National Institute of Science and Technology (UNIST), Ulsan, 689-798, Republic of Korea

**Keywords:** Phase separation, Silica nanosphere, Porous polymer membrane, Porous carbon

## Abstract

We successfully synthesized two different structures, silica nanospheres and porous polymer membranes, via nanophase separation, based on a sol–gel process. Silica sol, which was *in situ* polymerized from tetraorthosilicate, was used as a precursor. Subsequently, it was mixed with a polymer that was used as a matrix component. It was observed that nanophase separation occurred after the mixing of polymer with silica sol and subsequent evaporation of solvents, resulting in organizing various structures, from random network silica structures to silica spheres. In particular, silica nanospheres were produced by manipulating the mixing ratio of polymer to silica sol. The size of silica beads was gradually changed from micro- to nanoscale, depending on the polymer content. At the same time, porous polymer membranes were generated by removing the silica component with hydrofluoric acid. Furthermore, porous carbon membranes were produced using carbon source polymer through the carbonization process.

## Background

Considerable efforts have been devoted to the design and fabrication of controlled organic/inorganic composites with novel properties, including optical, electrical, chemical, biological, and mechanical properties [1-4]. In these hybrid systems, phase separation occurs naturally because they are composed of two materials with totally different chemical characteristics [5-7]. When domain formation is induced by phase transition, the compatibility and interaction between organic and inorganic components are key factors to determine the uniformity and nanostructures of the final objects [8-10]. These factors contributed not only to the size of the nanostructured inorganic materials, but also to their morphologies, which can have an effect on the ultimate properties.

The composites prepared by the sol–gel-based process compared with other strategies including surface modification and development of new routes [11,12] show the possibility of creating well-organized homogeneous inorganic structures in an organic matrix, resulting in obtaining the expected properties [13-17]. In particular, silica nanoparticles prepared by sol–gel were regarded as one of the most useful materials and were used in practical applications such as inorganic additives [18-22]. Nevertheless, the need for various sizes of silica nanoparticles with narrow size distribution has increased gradually for high technology applications.

Recently, membrane technologies have been established on a large scale, owing to the intensive results so far achieved [23-27]. A membrane refers to a separating structure serving as a selective barrier, and the unique property of membranes is to separate between two phases. For example, they separate air to remove carbon dioxide from natural gas and produce pure water from seawater via water treatment. Among the various materials (e.g., metals, ceramics, and composites) used for membranes, polymers are the most attractive materials because the permeability and selectivity of polymer can be adjustable and organized simply by solution processing [28-32]. Furthermore, Kim et al., reported the porous carbon membranes fabricated by self-assembly [33,34].

Herein, we prepared a series of silica/polymer composites using nanophase separation based on the sol–gel process. We controlled the ratio of polymer to silica sol for fabricating silica nanospheres and porous polymer membrane simultaneously. The micro- or nanostructures of silica were tuned by controlling a mixing ratio of polymer and silica. At the same time, nanoporous polymer structures, which were reversely replicated to silica spheres, were obtained. Both silica nanospheres and/or porous polymer membranes were produced by a selective removal method, such as calcination, and a chemical etching process. In addition, porous carbon membranes were transferred from polymer sources by carbonization.

## Methods

### Materials

Low molecular weight poly(methyl methacrylate) (PMMA) (*M*_w_ = 75 kg/mol) and high molecular weight PMMA (*M*_w_ = 350 kg/mol) were purchased from Polymer Source Inc. (Quebec, Canada) and Sigma-Aldrich Corporation (St. Louis, MO, USA), respectively. Polyacrylonitrile (PAN) (*M*_w_ = 150 kg/mol) was supplied by Sigma-Aldrich. Analytical grade tetraorthosilicate (TEOS), hydrochloric acid (HCl), tetrahydrofuran (THF), and N,N-dimethylformamide (DMF) were purchased from Sigma-Aldrich to synthesize silica sol. The hydrofluoric acid (HF) (J.T. Baker, Avantor Performance Materials, Center Valley, PA, USA) was diluted by deionized water before use.

### Preparation of polymer/silica solution

The TEOS precursor was mixed with a diluted HCl solution in a volume ratio of 6:2.3. The diluted HCl solution was obtained by mixing 0.02 mL of a concentrated HCl with 10 mL of deionized water. THF was added to the aqueous TEOS solution in a volume ratio of 3:1 and stirred for 2 h. This solution was subsequently mixed in a volume ratio of 1:1 with a 3-wt.% polymer solution (PMMA in THF and PAN in DMF) for 2 h.

### Synthesis of nanostructured silica and polymer membranes

The resulting homogeneous solution was cast into a Teflon container and dried in a vacuum oven at 60°C for 6 h. The solid samples were produced after the evaporation of all solvents. As-synthesized polymer/silica composites were treated in two different ways to selectively remove one of the components. Calcination proceeded at 500°C for 3 h in air condition to obtain pure silica particles. On the other hand, polymer membranes were prepared by immersing the samples in a diluted 5 wt.% HF solution and subsequently rinsed several times with deionized water. Porous carbon membranes were prepared by a carbonization process (850°C for 3 h in an argon environment) of PAN/silica composites. A scanning electron microscope (SEM) (NanoSEM 230, FEI Company, Hillsboro, OR, USA) operating at 10 kV was used to characterize the surface morphologies of as-prepared silica/polymer composites, nanostructured silica, and polymer membranes. Raman spectrum was recorded on a JASCO spectrometer (NRS 3000; JASCO Inc., Easton, MD, USA) to investigate the characteristics of carbon materials. An He-Ne laser was operated at *λ* = 632.8 nm.

## Results and discussion

**Figure 1:**
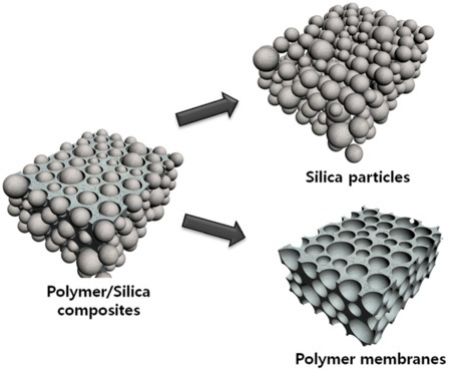
**Schematic illustration of silica spheres and polymer membranes produced by a selective etching process.** Polymer/silica composites, silica spheres, and porous polymer membranes can be prepared by phase separation between polymer and silica precursor.

The schematic illustration of Figure 1 shows the products of each step involving polymer/silica composites, silica nanospheres, and polymer membranes through polymer/silica mixing and selective removal processes. The polymer/silica hybrid structures were produced by uniformly dispersing the silica sol into the polymer solution and subsequent evaporation of the solvents. The resulting composites consist of micro- and/or nanostructured silica and a polymer matrix. Depending on the selective removal condition of one component, nanostructured silica was obtained by calcination process, while the polymer membrane was fabricated by a chemical etching process.

**Figure 2:**
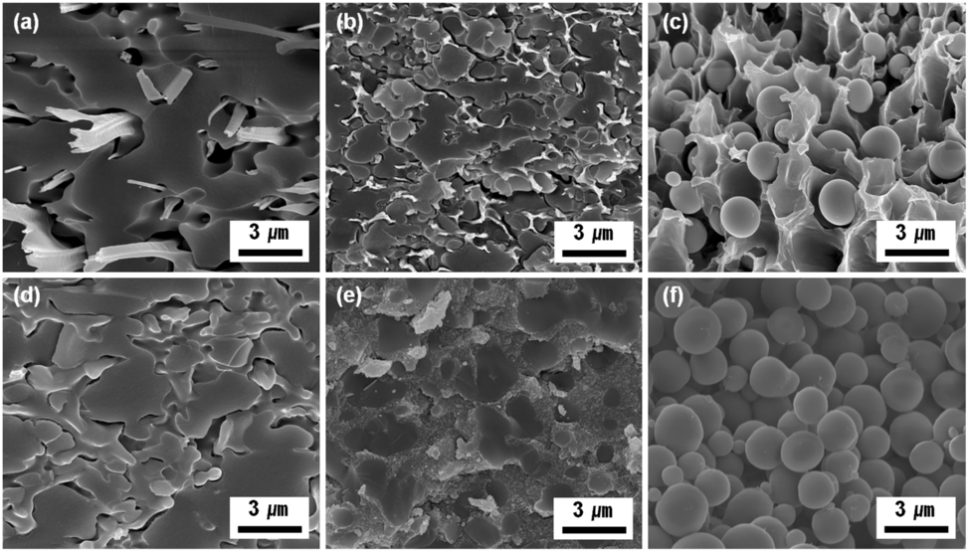
**SEM images of low molecular weight PMMA mixed with silica sol.** Mixing ratio of (**a**) 1:2, (**b**) 1:1, and (**c**) 2:1 of PMMA/silica sol, and (**d, e, f**) the corresponding silica structures of (**a, b, c**), after removing PMMA, respectively.

First, low molecular weight PMMA was used to synthesize the PMMA/silica composites with three different volumetric ratios. By controlling the mixing ratio of PMMA and silica sol, the surface morphologies of the composites were tuned from random network silica to spherical silica, as shown in Figure 2. Silica sol dispersed in the polymer solution formed the certain morphologies via phase separation, due to the incompatibility between silica and polymer, upon drying. When the contents of the silica components were higher than those of PMMA, minor PMMA was dispersed in the major silica matrix (Figure 2a). As the PMMA contents were increased to the same volumetric ratio with silica, silica particles started to be aggregated (Figure 2b). With a further increase of polymer (volumetric ratio of 2:1, PMMA/silica), the surface morphologies of silica/polymer composites were significantly transformed to silica microspheres (an average diameter of 1.6 μm), uniformly dispersed in the PMMA matrix (Figure 2c). The corresponding spatial locations of silica components were clearly investigated by calcination process in air condition, in which PMMA were completely degraded, while silica components were left over without changing the morphologies (Figure 2d,e,f). It should be noted that the morphologies of PMMA/silica composites could be simply controlled, from random network structure to spheres, by tuning the volumetric ratios between polymer and silica.

**Figure 3:**
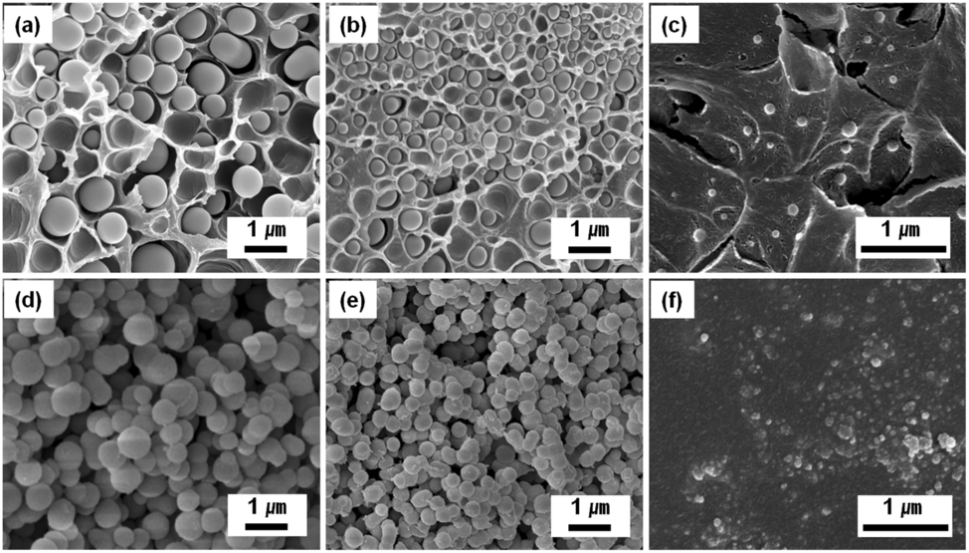
**SEM images of low molecular weight PMMA mixed with silica sol.** Mixing ratio of (**a**) 3:1, (**b**) 4:1, and (**c**) 5:1 of PMMA/silica sol, and (**d, e, f**) the corresponding silica beads of (**a, b, c**), after removing PMMA, respectively.

In order to reduce the size of silica sphere to the nanosized dimension, the volumetric ratio of PMMA to silica sol was further changed. With an increase of PMMA content, smaller silica spheres, with an average diameter of 590 nm, were synthesized at the ratio of polymer/silica = 3:1 (Figure 3a). In a similar manner, the silica nanospheres with the sizes of 345 and 77 nm were synthesized by increasing the PMMA contents to the ratio of 4:1 and 5:1, respectively (Figure 3b,c). These results indicated that the large amount of PMMA prevented the growth of silica sol during the nanophase separation. After removing the PMMA components, the shape and size of silica nanospheres were clearly observed by SEM, as shown in Figure 3d,e,f.

**Figure 4:**
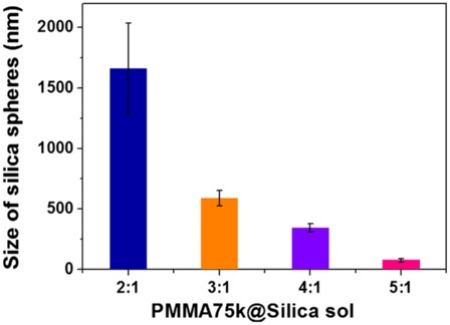
Histogram showing sizes of silica spheres as a function of mixing ratio of PMMA/silica sol.

The size of silica particles dispersed in the PMMA matrix, synthesized by phase separation, was presented in Figure 4. It was found that the size of a silica sphere strongly depended on the amounts of silica relative to the PMMA contents. Silica microspheres were synthesized in the volumetric ratio of 2:1 (polymer-to-silica sol). With the increasing polymer amounts, microscale silica was reduced to the nanoscale silica spheres with narrower size distribution.

**Figure 5:**
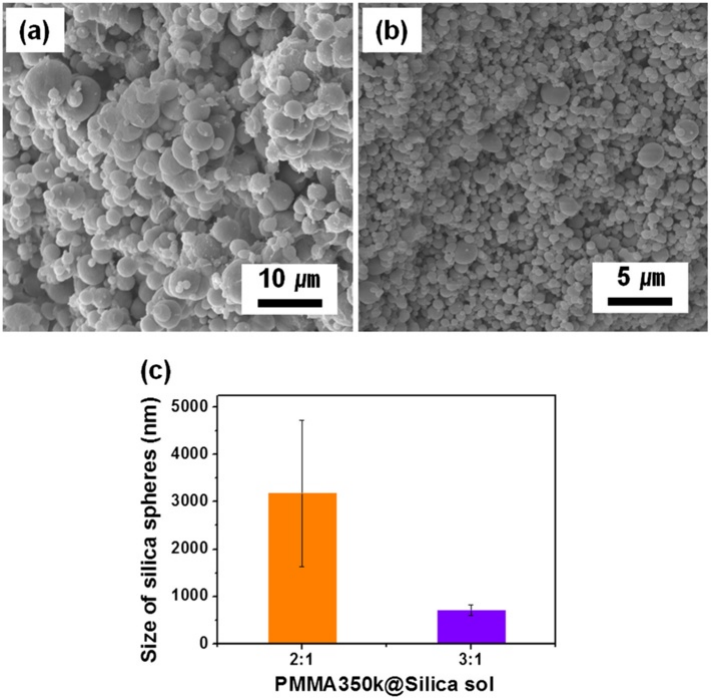
**SEM images of high molecular weight PMMA mixed with silica sol.** Mixing ratio of (**a**) 2:1, (**b**) 3:1 of PMMA/silica sol, and (**c**) the histogram showing the sizes of the corresponding samples.

We extended this idea to the high molecular weight PMMA (350 kg/mol) system. Figure 5 shows SEM images of silica particles synthesized by PMMA/silica composites with volumetric ratios of 2:1 and 3:1. After removal of PMMA via calcination process, silica spheres with average diameters of 3.2 μm and 710 nm were obtained from the composites with ratios of 2:1 and 3:1, respectively (Figure 5a,b). In the case of high molecular weight PMMA, there are some differences, compared to the low molecular weight system. Notably, the size of as-synthesized silica particles is larger than that prepared from low molecular weight PMMA. The other difference is that the size distribution of spheres is much broader in the high molecular weight system (Figure 5c). This is because phase separation between higher molecular weight PMMA and silica sol took place much faster than that of the lower molecular weight system. Also, the long polymer chains may prevent the mobility of silica sol that tends to be well dispersed in the polymer matrix. It should be noted that the combination of smaller molecular weight polymer and silica sol is a more efficient way to synthesize uniform micro- and/or nanosized silica spheres.

**Figure 6:**
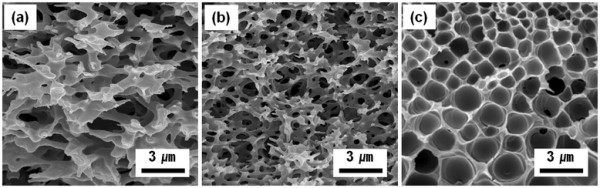
**SEM images of PMMA membranes obtained by a selective chemical etching of PMMA/silica.** Samples with mixing ratio of (**a**) 1:2, (**b**) 1:1, and (**c**) 2:1 (PMMA/silica sol) seen in Figure 2 were used.

In addition to synthesis of silica spheres, the polymer matrix in the polymer/silica composites can be left over to etch selective silica particles. Figure 6 shows SEM images of PMMA membranes that were obtained from PMMA/silica composites, by the selective removal of silica via a chemical etching in aqueous HF solution. When the samples (polymer/silica = 1:2) seen in Figure 2a were immersed in 5 wt.% HF solution, network-like silica structures were selectively removed, while PMMA layered structures with macropores were observed (Figure 6a).

When the sample seen in Figure 2b that has increasing polymer content was employed, PMMA membranes with smaller pore size were fabricated. In a similar manner, PMMA membranes of Figure 6c with uniform pores can be prepared from the samples seen in Figure 2c. Morphologies of polymer membranes seen in Figure 6 are the same as the replicated silica structures seen in Figure 2. Depending on the applications, nanostructured silica and/or polymer membranes can be selectively left over or removed.

**Figure 7:**
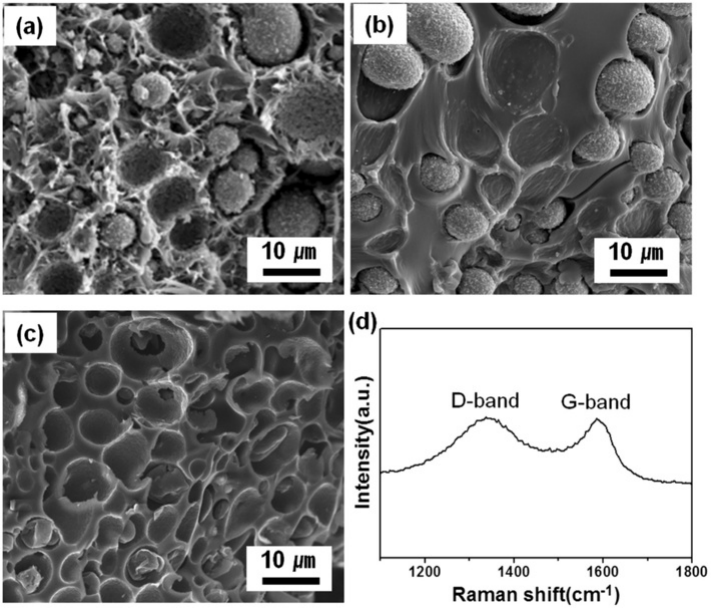
**SEM images of the fabricated structures.** (**a**) PAN/silica composites with a mixing ratio of 2:1, (**b**) carbon/silica prepared by carbonization process, (**c**) macroporous carbon membranes obtained by selective etching of silica, and (**d**) Raman spectrum of the porous carbon material showing an amorphous carbon structure (*I*_D_/*I*_G_ = 2.2).

Moreover, porous carbon membranes can be produced by combining PAN, one of the good carbon source materials, with silica sol. The PAN/silica composites were successfully synthesized at a ratio of 2:1 in DMF solution (Figure 7a). The silica microspheres were uniformly dispersed in the PAN matrix, according to similar mechanisms as those of PMMA/silica composites. However, the size of the silica sphere (approximately 15 μm) was significantly increased compared with that of the PMMA/silica system. It is attributed to the enhanced incompatibility between PAN and silica sol. When the PAN/silica composites were carbonized at 850°C for 3 h in an argon environment, the silica sphere remained unchanged, while the PAN was transformed to amorphous carbon without changing the spatial locations (Figure 7b). Subsequently, when the silica was selectively removed in an HF solution, porous carbon membranes were successfully achieved (Figure 7c). Raman scattering of the porous carbon shows two peaks at approximately 1,360 and approximately 1,580 cm^−1^ corresponding to the disordered band (D band) and the graphene (G band), respectively. The ratio of the D band to the G band was estimated to be 2.2, indicating an amorphous carbon structure (Figure 7d).

## Conclusions

We have successfully synthesized uniform-sized silica spheres and porous polymer membranes using a concept of nanophase separation. Incompatibility between polymer and silica sol induced the nanophase separation, resulting in the formation of polymer/silica composites. In this manner, the size of silica spheres could be tuned in the range of 1.6 μm to 80 nm by controlling the mixing ratio of polymer to silica sol after calcination process. Concurrently, a selective chemical etching of the same polymer/silica composites led to the formation of porous polymer membranes. Moreover, when polymer that can be used as a carbon source was used to make polymer/silica composites, followed by a chemical etching in HF solution, macroporous carbon membranes were successfully fabricated. This simple but straightforward process can be used in other applications, such as photonic bandgap, antireflection coating, lithium-ion batteries, and so on.

## Competing interests

The authors declare that they have no competing interests.

## Authors’ contributions

JL carried out experiments concerning phase separation of polymer and silica precursor and drafted the manuscript. SC carried out experiments concerning synthesis of porous carbon materials. SP designed this work and prepared the manuscript. All authors read and approved the final manuscript.

## References

[B1] Imazato S Antibacterial properties of resin composites and dentin bonding systems Dent Mater 2003 19 449 10.1016/S0109-5641(02)00102-1 12837391

[B2] Bledzki AK Gassan J Composites reinforced with cellulose based fibres Prog Polym Sci 1999 24 221 10.1016/S0079-6700(98)00018-5

[B3] Stankovich S Dikin DA Dommett GHB Kohlhaas KM Zimney EJ Stach EA Piner RD Nguyen ST Ruoff RS Graphene-based composite materials Nature 2006 442 282 10.1038/nature04969 16855586

[B4] Qiao Y Bao S-J Li CM Cui X-Q Lu Z-S Guo J Nanostructured polyaniline/titanium dioxide composite anode for microbial fuel cells ACS Nano 2008 2 113 10.1021/nn700102s 19206554

[B5] Zhang Q Archer LA Poly(ethylene oxide)/silica nanocomposites structure and rheology Langmuir 2002 18 10435 10.1021/la026338j

[B6] Nakanishi K Pore structure control of silica gels based on phase separation J Porous Mater 1997 4 67 10.1023/A:1009627216939

[B7] Lipatov YS Nesterov AE Ignatova TD Nesterov DA Effect of polymer–filler surface interactions on the phase separation in polymer blends Polymer 2002 43 875 10.1016/S0032-3861(01)00632-2

[B8] Fu S-Y Feng X-Q Lauke B Mai Y-W Effects of particle size, particle/matrix interface adhesion and particle loading on mechanical properties of composites Composites: Part B 2008 39 933 10.1016/j.compositesb.2008.01.002

[B9] Goltner-Spickermann C Non-ionic templating of silica: formation mechanism and structure Curr Opin Colloid & Interface Sci 2002 7 173 10.1016/S1359-0294(02)00046-8

[B10] Park C Hyun DC Lim MC Kim SJ Kim YR Paik HJ Jeong U Continuous production of functionalized polymer particles employing the phase separation in polymer blend films Macromol Rapid Commun 2011 32 1247 10.1002/marc.201100199 21648009

[B11] Caruso F Nanoengineering of particle surfaces Adv Mater 2001 13 11 10.1002/1521-4095(200101)13:1<11::AID-ADMA11>3.0.CO;2-N

[B12] Hu J Chen M Wu L Organic–inorganic nanocomposites synthesized via miniemulsion polymerization Polym Chem 2011 2 760 10.1039/c0py00284d

[B13] Sun J Akdogan EK Klein LC Safari A Characterization and optical properties of sol–gel processed PMMA-SiO2 hybrid monoliths J Non-Cryst Solids 2007 353 2807 10.1016/j.jnoncrysol.2007.05.158

[B14] Yeh J-M Hsieh C-F Yeh C-W Wu M-J Yang H-C Organic base-catalyzed sol–gel route to prepare PMMA-silica hybrid materials Polym Int 2007 56 343 10.1002/pi.2143

[B15] Zou H Wu S Shen J Polymer/silica nanocomposites: preparation, characterization, properties, and applications Chem Rev 2008 108 3893 10.1021/cr068035q 18720998

[B16] Morikawa A Lyoku Y Kakimoto M Mai Y Preparation of new polyimide-silica hybrid materials via the sol–gel process J Mater Chem 1992 2 679 10.1039/jm9920200679

[B17] Caruso RA Antonietti M Sol–gel nanocoating: an approach to the preparation of structured materials Chem Mater 2001 13 3272 10.1021/cm001257z

[B18] Lu Y Yin Y Mayers BT Xia Y Modifying the surface properties of superparamagnetic iron oxide nanoparticles through a sol–gel approach Nano Lett 2002 2 183 10.1021/nl015681q

[B19] Bogush GH Tracy MA Zukoski CF Preparation of monodisperse silica particles: control of size and mass fraction J Non-Cryst Solids 1988 104 95 10.1016/0022-3093(88)90187-1

[B20] Barbe C Bartlett J Kong L Finnie K Lin HQ Larkin M Calleja S Bush A Calleja G Silica particles: a novel drug-delivery system Adv Mater 2004 16 1959 10.1002/adma.200400771

[B21] Rosenholm JM Sahlgren C Linden M Towards multifunctional, targeted drug delivery systems using mesoporous silica nanoparticles – opportunities & challenges Nanoscale 1870 2010 2 10.1039/c0nr00156b 20730166

[B22] Stöber W Fink A Controlled growth of monodisperse silica spheres in the micron size range J Colloid Interface Sci 1968 26 62 10.1016/0021-9797(68)90272-5

[B23] Ulbricht M Advanced functional polymer membranes Polymer 2006 47 2217 10.1016/j.polymer.2006.01.084

[B24] Freeman BD Basis of permeability-selectivity tradeoff relations in polymeric gas separation membranes Macromolecules 1999 32 375 10.1021/ma9814548

[B25] Sokalski T Ceresa A Zwickl T Pretsch E Large improvement of the lower detection limit of ion-selective polymer membrane electrodes J Am Chem Soc 1997 119 11347 10.1021/ja972932h

[B26] Tanaka M Sackmann E Polymer-supported membranes as models of the cell surface Nature 2004 437 656 16193040 10.1038/nature04164

[B27] Lee K-S Jeong M-H Lee J-P Kim Y-J Lee J-S Synthesis and characterization of highly fluorinated cross-linked aromatic polyethers for polymer electrolytes Chem Mater 2010 22 5500 10.1021/cm101405h

[B28] Li Q Jensen JO Savinell RF Bjerrum NJ High temperature proton exchange membranes based on polybenzimidazoles for fuel cells Prog Polym Sci 2009 34 449 10.1016/j.progpolymsci.2008.12.003

[B29] Ham H Chung I Choi Y Lee S Kim S Macroporous polymer thin film prepared from temporarily stabilized water-in-oil emulsion J Phys Chem B 2006 110 13959 10.1021/jp0616361 16836347

[B30] Bakker E Buhlmann P Pretsch E Polymer membrane ion-selective electrodes-What are the limits? Electroanalysis 1999 11 915 10.1002/(SICI)1521-4109(199909)11:13<915::AID-ELAN915>3.0.CO;2-J

[B31] Park J Lee S Han T Kim S Hierarchically ordered polymer films by templated organization of aqueous droplets Adv Funct Mater 2007 17 2315 10.1002/adfm.200601141

[B32] Widawski G Rawiso M Francois B Self-organized honeycomb morphology of star-polymer polystyrene films Nature 1994 369 387 10.1038/369387a0

[B33] Lee S Park J Lim B Mo C Lee W Lee J Hong S Kim S Highly entangled carbon nanotube scaffolds by self-organized aqueous droplets Soft Matter 2009 5 2343 10.1039/b817477f

[B34] Lee S Kim H Hwang J Lee W Kwon J Bielawski C Ruoff R Kim S Three-dimensional self-assembly of graphene oxide platelets into mechanically flexible macroporous carbon films Angew Chem Int Ed 2010 49 10084 10.1002/anie.201006240 21117056

